# Processing, Export, and Identification of Novel Linear Peptides from Staphylococcus aureus

**DOI:** 10.1128/mBio.00112-20

**Published:** 2020-04-14

**Authors:** Katrin Schilcher, Lindsay K. Caesar, Nadja B. Cech, Alexander R. Horswill

**Affiliations:** a Department of Immunology and Microbiology, University of Colorado School of Medicine, Aurora, Colorado, USA; b Department of Chemistry and Biochemistry, University of North Carolina at Greensboro, North Carolina, USA; c Department of Veterans Affairs Eastern Colorado Health Care System, Denver, Colorado, USA; University of Rochester

**Keywords:** *S. aureus*, linear peptides, lipoproteins, Eep, EcsAB, *Staphylococcus aureus*

## Abstract

Staphylococcus aureus can colonize the human host and cause a variety of superficial and invasive infections. The success of S. aureus as a pathogen derives from its ability to modulate its virulence through the release, sensing of and response to cyclic signaling peptides. Here we provide, for the first time, evidence that S. aureus processes and secretes small linear peptides through a specialized pathway that converts a lipoprotein leader into an extracellular peptide signal. We have identified and confirmed the machinery for each step and demonstrate that the putative membrane metalloprotease Eep and the EcsAB transporter are required to complete the processing and secretion of the peptides. In addition, we have identified several linear peptides, including the interspecies signaling molecule *staph*-cAM373, that are dependent on this processing and secretion pathway. These findings are particularly important because multiple Gram-positive bacteria rely on small linear peptides to control bacterial gene expression and virulence.

## Abstract (precis)

Here, we provide evidence indicating that S. aureus secretes small linear peptides into the environment via a novel processing and secretion pathway. The discovery of a specialized pathway for the production of small linear peptides and the identification of these peptides leads to several important questions regarding their role in S. aureus biology, most interestingly, their potential to act as signaling molecules. The observations in this study provide a foundation for further in-depth studies into the biological activity of small linear peptides in S. aureus.

## OBSERVATION

Staphylococcus aureus colonizes approximately a third of the human population and is the most common cause of skin and soft tissue infections in otherwise healthy community individuals (reviewed in references 1 and 2). S. aureus virulence is controlled by numerous regulatory systems, one of which is a quorum sensing system that responds to cyclic peptide signals (reviewed in references 3 and 4). Other Gram-positive bacteria have evolved small linear peptide signaling systems to control a variety of critical processes (reviewed in references 5 and 6). Enterococcus faecalis uses small linear peptides (7 to 8 amino acids in length) that induce a mating response of donor cells carrying cognate conjugative plasmids (7). Interestingly, S. aureus produces the hydrophobic, linear heptapeptide *staph*-cAM373 (amino acids AIFILAA), which mimics the enterococcal cAM373 peptide (AIFILAS) by mediating aggregation of E. faecalis cells harboring the pAM373 plasmid and subsequent interspecies horizontal gene transfer (HGT) *in vitro* (8–10). Interspecies HGT is of special interest, because vancomycin resistance in S. aureus has been proposed to have occurred through HGT from vancomycin-resistant E. faecalis (11).

Small linear peptides often derive from the secretion signal sequences of lipoprotein precursors that contain an N-terminal sequence with a conserved C-terminal lipobox motif. After predominantly Sec-dependent secretion, the lipoprotein is processed and attached to the membrane via the conserved phosphatidylglycerol prolipoprotein diacylglyceryl transferase (Lgt) and the specific signal peptidase II (LspA) (reviewed in references 12 and 13). In addition to the upstream processing events, the intramembrane metalloprotease RseP/Eep and the EcsAB/PptAB transporter were shown to be involved in linear peptide processing and secretion in diverse bacterial species (14–17). This is especially intriguing because the *ecsAB* locus is conserved in S. aureus (18) and shows high homology to the enterococcal proteins (60% and 30% amino acid identity for EcsA and EcsB, respectively), while Eep shares 39% amino acid identity with the enterococcal metalloprotease RseP/Eep. However, the substrates for both membrane proteins and the machinery responsible for processing and export of linear peptides in S. aureus are undefined.

**FIG 1:**
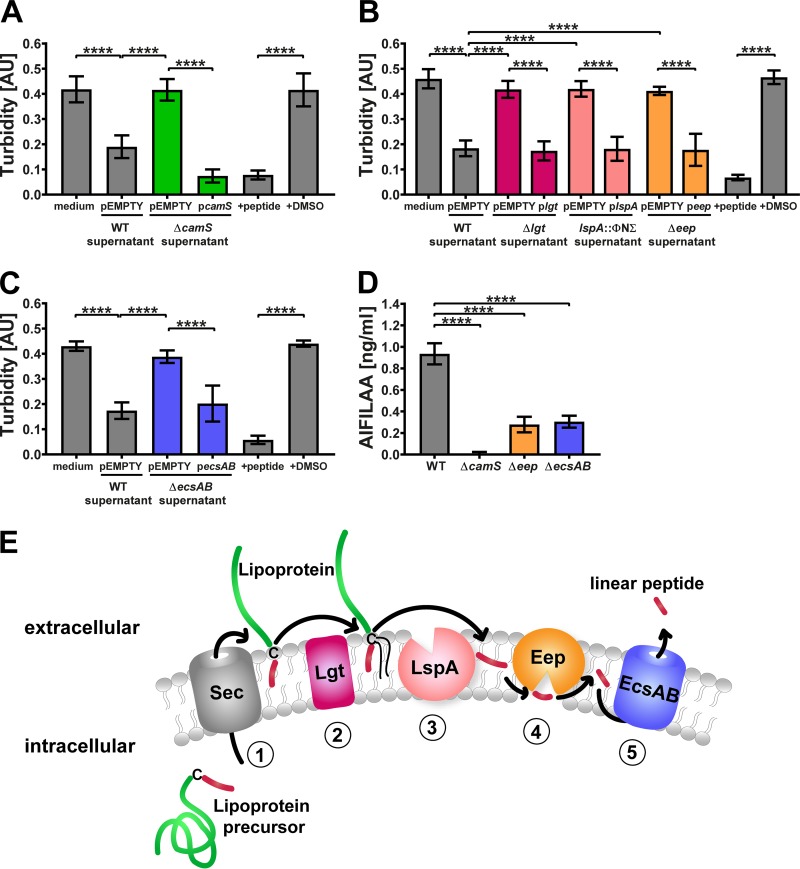
E. faecalis JH2-2 pAM373::Tn*918* aggregates in response to supernatant of the S. aureus USA300 LAC wild-type (WT) but not the isogenic (A) *camS* (Δ*camS*); (B) *lgt* (Δ*lgt*), *lspA* (*lspA*::ΦNΣ), and *eep* (Δ*eep*); and (C) *ecsAB* (Δ*ecsAB*) mutants. Mutant phenotypes were complemented to the WT level with the corresponding genes expressed on plasmids in the respective mutant backgrounds. Addition of 20 nM synthetic *staph*-cAM373 but not the peptide solvent DMSO stimulated E. faecalis aggregation. pEMPTY = empty expression vector. (*n* = 5 biological replicates, means ± standard deviations [SD].) AU, arbitrary units. (D) Quantification of *staph*-cAM373 in cell-free supernatant of LAC WT and *camS*, *eep*, and *ecsAB* mutant background by mass spectrometry. Shown is the absolute quantification of the *staph*-cAM373 peptide (retention time 4.56 min, measured *m/z* 718.4504) using a calibration curve of a synthetic peptide standard (retention time 4.56, *m/z* 718.4492) (*n* = 5 biological replicates, means ± SD). ****, *P ≤ *0.0001. (E) Proposed model of linear peptide processing and export in S. aureus. The lipoprotein precursor is secreted through the secretory apparatus (step 1) and then processed via Lgt (step 2) and LspA (step 3). Peptide maturation involves Eep (step 4) and subsequent export via EcsAB (step 5) outside the cell.

We aimed to identify the molecular determinants of linear peptide processing and export in the community-acquired methicillin-resistant S. aureus (MRSA) USA300 LAC strain. To identify candidate genes, we employed an E. faecalis aggregation assay to assess the production of the linear peptide *staph*-cAM373 in S. aureus culture supernatant. Briefly, E. faecalis cells harboring the pAM373 plasmid respond to the presence of *staph*-cAM373 by formation of cellular aggregates (8). Exogenous addition of synthetic *staph*-cAM373 peptide (AIFILAA) or LAC wild-type (WT) supernatant resulted in aggregation of E. faecalis pAM373 cells and low culture turbidity (Fig. 1A to C). In contrast, supernatant of the isogenic *camS* mutant strain, which lacks the lipoprotein and *staph*-cAM373, resulted in a significant reduction of E. faecalis aggregation accompanied by higher turbidity (Fig. 1A). The loss of the aggregation phenotype was also observed with supernatants from the *lgt* and *lspA* mutants (Fig. 1B). Most interestingly, mutation of *eep* and *ecsAB* inhibited the aggregation phenotype of E. faecalis pAM373 to the same degree as that observed with the *camS* mutant (Fig. 1B and C). Complementation of the mutated genes on a plasmid restored the aggregation phenotype for each of the five mutant strains to WT levels. To confirm the importance of Eep and EcsAB for *staph*-cAM373 production, we analyzed supernatant of the LAC WT strain as well as the *camS*, *eep*, and *ecsAB* mutant strains by high-resolution mass spectrometry (MS). As expected, *staph*-cAM373 was present in the supernatant of the WT but not in that of the *camS* mutant (Fig. 1D). Importantly, we detected only minimal levels of *staph*-cAM373 in the supernatant of the *eep* and *ecsAB* mutant strains, confirming their importance for *staph*-cAM373 processing and export. Taken together, we can now model the processing and secretion pathway of the interspecies linear peptide signal *staph*-cAM373 (Fig. 1E).

**FIG 2:**
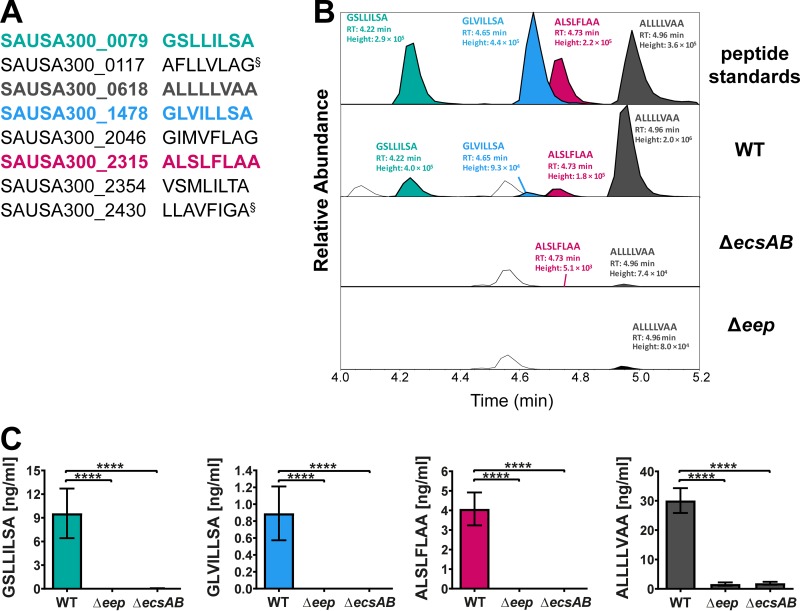
(A) Identified linear peptides in LAC WT. The symbol § indicates peptides that share the same accurate mass, therefore, the identity of the detected ion could not be resolved. (B) Schematic diagram of the overlaid LC-MS profiles of the four linear peptides found in LAC WT. In the top panel, all peptides are shown at a concentration of 10 ng/ml. All peaks highlighted in the bacterial strains have been normalized to the peak height of the maximum detected peak (1.95 × 10^6^) to illustrate differences in production within and between strains. Peaks that have not been colored in represent isobars of peptides under study. (C) Production of all four linear peptides is significantly reduced in the *eep* (Δ*eep*) and *ecsAB* (Δ*ecsAB*) mutant strains. Shown are the absolute quantifications of all four peptides determined using a calibration curve of the corresponding synthetic peptide standard for each peptide (*n* = 5 biological replicates, means ± SD). ****, *P ≤ *0.0001.

To determine whether Eep and EcsAB are components of a global S. aureus linear peptide biosynthetic pathway, we mined the peptidome data obtained from the LAC WT strain for the presence of additional linear peptides derived from the secretion signal sequence of reported staphylococcal lipoproteins (19) with characteristics similar to those of *staph*-cAM373. Ions with masses matching (mass error < 5 ppm) those of eight peptides were detected in the supernatant of the LAC WT strain but not in the *eep* and *ecsAB* mutant strains (Fig. 2A; see also Table S1 in the supplemental material). We confirmed the presence of four of the putative linear peptides in the LAC WT supernatant by comparing their *m/z* values and retention times to those of synthetic standards (Fig. 2B). Subsequently, we also quantified these four linear peptides in the peptidome of the *eep* and *ecsAB* mutant strains. All four peptides were either completely absent or present only at very low levels compared to the WT strain (Fig. 2B and C). These data clearly demonstrate that Eep and EcsAB have global importance for linear peptide signal production in S. aureus.

10.1128/mBio.00112-20.4 TABLE S1 Identified linear peptides in S. aureus. Download Table S1, DOCX file, 0.01 MB. Copyright © 2020 Schilcher et al. 2020 Schilcher et al. This content is distributed under the terms of the Creative Commons Attribution 4.0 International license.

Taking the results together, we used a combination of genome mining, genetic characterization, and mass spectrometry to model the processing and secretion pathway of the interspecies linear peptide signal *staph*-cAM373 and to demonstrate that Eep and EcsAB are of global importance for linear peptide signal production in S. aureus. Whereas many of the peptides identified by our approach derive from uncharacterized proteins, the *staph*-cAM373 linear peptide is a known interspecies signaling peptide (8–10), suggesting that Eep and EcsAB are likely to be additional important determinants for HGT. Two of the Eep-dependent and EcsAB-dependent peptides derive from lipoproteins (SAUSA300_0079 and SAUSA300_0618) involved in metal binding (20, 21). Thus, Eep and EcsAB might be of importance for survival when S. aureus encounters host-imposed metal stress during infection. With our new knowledge of the pathway for linear peptide processing and secretion, in-depth studies on the biological function of linear peptides in S. aureus can be explored.

## 

### Methods used.

Descriptions of methods used for generation of gene deletions and complementations including supplemental references are provided in Text S1. Bacterial strains/plasmids are listed in Table S2 and primers are listed in Table S3.

10.1128/mBio.00112-20.1 TEXT S1 Supplemental materials and methods. Download Text S1, DOCX file, 0.03 MB. Copyright © 2020 Schilcher et al. 2020 Schilcher et al. This content is distributed under the terms of the Creative Commons Attribution 4.0 International license.

### Reagents and growth conditions.

Peptides were custom synthesized by AnaSpec Inc. (Fremont, CA) and resuspended in dimethyl sulfoxide (DMSO) at a concentration of 1 mg/ml. All bacterial strains and plasmids used in this work are listed in Table S2. Escherichia coli was cultured in lysogeny broth (LB), and for most experiments, S. aureus and E. faecalis were cultured in tryptic soy broth (TSB) at 37°C with shaking at 225 rpm. For assessment of aggregation and mass spectrometry analysis, S. aureus strains were grown in chemically defined medium (CDM), prepared as described by Ibberson et al. (22). Antibiotics for S. aureus selection and plasmid maintenance were added to the media at the following concentrations: chloramphenicol (Cm), 10 μg/ml; erythromycin (Erm), 5 μg/ml. E. coli plasmids were maintained in media supplemented with ampicillin (Amp) (100 μg/ml) or chloramphenicol (Cm) (10 μg/ml). Tetracycline (Tet) was added at 10 μg/ml to E. faecalis cultures when necessary.

10.1128/mBio.00112-20.5 TABLE S2 Bacterial strains/plasmids. Download Table S2, DOCX file, 0.02 MB. Copyright © 2020 Schilcher et al. 2020 Schilcher et al. This content is distributed under the terms of the Creative Commons Attribution 4.0 International license.

### S. aureus supernatant preparation for cell aggregation and mass spectrometry.

S. aureus overnight cultures were grown in CDM and diluted to an optical density at 600 nm (OD_600_) of 0.02 in 5 ml fresh CDM and grown at 37°C with shaking (225 rpm) to an OD_600_ of 2.8 to 3. Cells were harvested by centrifugation, and the supernatant was filtered through a 0.22-μm-pore-size filter (Millipore; Millex polyethersulfone [PES] membrane). Proteinase inhibitor cocktail (SigmaFAST, EDTA free; Sigma) was added to the supernatant, and the samples were stored at 4°C.

### Cell aggregation assay.

E. faecalis cell aggregation was assayed using a protocol modified from that previously reported by Dunny et al. (7). E. faecalis JH2-2 cells containing peptide-responsive pAM373::Tn*918* plasmid were grown overnight in TSB at 37°C with 225 rpm shaking, washed twice, and resuspended to an OD_600_ of 0.2 (9.1E + 07 ± 6.2E + 06 CFU per ml) in CDM. S. aureus supernatant (1 ml) was mixed with 1 ml of E. faecalis cells in a round-bottom glass tube (Kimax; Kimble) and incubated at 37°C in a shaking incubator (250 rpm) for 4.5 h. Cell aggregation was quantified by measuring the turbidity of the suspension with a MicroScan turbidity meter (Dade Behring) compared to that of the medium. To control for E. faecalis growth effects, cell aggregates were dissolved by addition of 2 mM EDTA followed by vortex mixing of the suspension for 2 min. Subsequently, turbidity was determined as described above and bacteria were plated on tryptic soy agar (TSA) to determine CFU counts per ml (see Fig. S1A and B in the supplemental material).

10.1128/mBio.00112-20.2 FIG S1 (A) Turbidity measurement of E. faecalis JH2-2 pAM373::Tn*918* grown in the presence of medium or cell-free supernatant of the S. aureus wild-type (WT) or mutants (Δ*camS*, Δ*lgt*, *lspA*::ΦNΣ, Δ*eep* and Δ*ecsAB*) carrying pEMPTY. Cultures were measured before (black bars) and after (gray bars) addition of 2 mM EDTA and vortex mixing to disperse cell aggregates. AU, arbitrary units. (B) Viable cell counts (CFU/ml) of E. faecalis JH2-2 pAM373::Tn*918* cells after dispersal with 2 mM EDTA and vortex mixing (*n* ≥ 3 biological replicates, means ± SD). Download FIG S1, TIF file, 1.0 MB. Copyright © 2020 Schilcher et al. 2020 Schilcher et al. This content is distributed under the terms of the Creative Commons Attribution 4.0 International license.

### Mass spectrometry analysis.

All liquid chromatography-mass spectrometry (LC-MS) analyses were conducted using a Waters Acquity ultraperformance LC (UPLC) system (Waters Corporation) coupled to a Thermo Fisher Scientific Q-Exactive Plus mass spectrometer (Thermo Fisher Scientific) with electrospray ionization (ESI). Samples (3 μl) were injected into a Waters BEH C_18_ column (1.7-μm pore size; 2.1 by 50mm) at a flow rate of 0.3 ml/min with a gradient of H_2_O (solvent A) and acetonitrile (solvent B), each spiked with 0.1% formic acid. The gradient began at 5% solvent B for 0.5 min and increased to 40% solvent B using a gradient curve of 5 from 0.5 to 7.5 min. From 7.5 to 8.0 min, the gradient linearly increased to 100% solvent B and was held at 100% solvent B for 0.5 min. The starting conditions were reestablished from 8.5 to 9.0 min and held until 10.0 min.

Analyses were conducted in positive-ion and negative-ion modes with a scan range of *m/z* 175 to 2,000, spray voltage of 3.7 kV, capillary temperature of 265.63°C, sheath gas flow of 51.25, auxiliary gas flow of 13.13, and S-lens radio frequency (RF) level of 80.00. Peptide concentrations were calculated using calibration curves of synthetic peptides ranging from 0 to 100 ng/ml by 2-fold dilutions (Fig. S2A). Calibration curves were generated with positive-mode data by plotting the peak area versus the concentration, and the linear portion of each curve was determined by log transformation of both axes. Peak areas were selected for *m/z* values of 783.5330, 805.4812, 773.4774, 785.5125, and 718.4492, corresponding to ALLLLVAA, ALSLFLAA, GSLLILSA, GLVILLSA, and AIFILAA, respectively. Concentrations were calculated using the best-fit line obtained by linear regression of each data set. Ions whose peak areas were below the detection limit were assigned a concentration of 0. Concentrations were normalized to CFU counts (Fig. S2B).

10.1128/mBio.00112-20.3 FIG S2 (A) Standard calibration curves of synthetic *staph*-cAM373 (AIFILAA) and four additional linear peptides derived from mass spectrometric measurements. The amino acid sequence of each peptide is indicated below the *x* axis in each plot. (B) Viable cell counts (CFU/ml) from the WT and *camS* (Δ*camS*), *eep* (Δ*eep*), and *ecsAB* (Δ*ecsAB*) mutant cultures used for the preparation of supernatant for peptide quantification (*n* = 5 biological replicates, means ± SD). Download FIG S2, TIF file, 2.8 MB. Copyright © 2020 Schilcher et al. 2020 Schilcher et al. This content is distributed under the terms of the Creative Commons Attribution 4.0 International license.

### Statistical analysis.

Statistical significance was calculated with GraphPad Prism (8.2.0) using a one-way analysis of variance (ANOVA) with a Tukey's or Dunnett's posttest to correct for multiple comparisons.

10.1128/mBio.00112-20.6 TABLE S3 Primer list. Download Table S3, DOCX file, 0.02 MB. Copyright © 2020 Schilcher et al. 2020 Schilcher et al. This content is distributed under the terms of the Creative Commons Attribution 4.0 International license.
