# Supplementary material for: Processing, Export, and Identification of Novel Linear Peptides from Staphylococcus aureus
Source: mBio. 2020 Apr 14;11(2):e00112-20. doi: 10.1128/mBio.00112-20 (PMC7157817; doi:10.1128/mBio.00112-20)
Supplement: TABLE S1 [file mBio.00112-20-st001.docx]

**Table S1:** Identified linear peptides in *S. aureus*

| **Signal sequence** | **detected putative peptide sequence** | **accurate mass ([M+H]+)** | **Retention Time** |
| --- | --- | --- | --- |
| MIKKLFFMILGSLLILSA | GSLLILSA* | 773.4774 | 4.23 min |
| MNKVIKMLVVTLAFLLVLAG | AFLLVLAG^§^ | 803.5032 | 5.02 min |
| MKKLVPLLLALLLLVAA | ALLLLVAA* | 783.5344 | 4.96 min |
| MRRWFVLILGLVILLSA | GLVILLSA* | 785.5137 | 4.65 min |
| MKKKALLPLFLGIMVFLAG | GIMVFLAG | 807.4439 | 4.67 min |
| MKRLVTGLLALSLFLAA | ALSLFLAA* | 805.4824 | 4.73 min |
| MTKKLLTLFIVSMLILTA | VSMLILTA | 847.4963 | 4.33 min |
| MMIHSKRLRLWLYLVLLAVFIGA | LLAVFIGA^§^ | 803.5032 | 5.02 min |

***** The presence of these peptides in one or more bacterial strains has been confirmed by comparing LC-MS data to those of purchased standards.

^§^ These peptides have the same accurate mass. Since the presence of these peptides was not confirmed by comparison to standard compounds, the identity of the detected ion at 5.02 min could not be resolved.
