# Supplementary material for: Processing, Export, and Identification of Novel Linear Peptides from Staphylococcus aureus
Source: mBio. 2020 Apr 14;11(2):e00112-20. doi: 10.1128/mBio.00112-20 (PMC7157817; doi:10.1128/mBio.00112-20)
Supplement: TEXT S1 [file mBio.00112-20-s0001.docx]

Supplemental Material and Methods for

**Processing, Export and Identification of Novel Linear Peptides from *Staphylococcus aureus***

Katrin Schilcher, Lindsay K. Caesar, Nadja B. Cech, Alexander R. Horswill

Alexander R. Horswill and Katrin Schilcher

Email: [alexander.horswill@cuanschutz.edu](mailto:alexander.horswill@cuanschutz.edu), [katrin.schilcher@cuanschutz.edu](mailto:katrin.schilcher@cuanschutz.edu)

**Material and Methods**

**Generation of gene deletions and complementations**

To construct markerless deletions of *camS* (SAUSA300_1884), *lgt* (SAUSA300_0744), *eep* (SAUSA300_1155) and the *ecsAB* operon (SAUSA300_1785, SAUSA300_1786), regions flanking the respective gene were amplified from USA300 LAC genomic DNA using primers (IDT, Coralville, IA) listed in Table S3. The amplified products were column purified (Qiagen), digested with restriction enzymes (NEB) and ligated into pJB38 or pIMAY to generate CFS246, pKAS07, pKAS22 and pKAS24 (Table S2). All plasmids were electroporated into *E. coli* DC10B, sequenced (primers listed in Table S3) at the Molecular Biology Service Center at the University of Colorado Anschutz Medical Campus and subsequently electroporated into LAC (AH1263). Deletions were generated as described in (1) for pJB38 and in (2, 3) for pIMAY and mutants were confirmed by PCR with primers listed in Table S3. The *lspA* mariner-based transposon *bursa aurealis* mutation (JE2 *lspA*::ΦNΣ, NE1757) from the Nebraska Transposon Library (4) was moved into LAC (AH1263) with phage 11 as described previously (5). *S. aureus* genomic DNA of LAC *lspA*::ΦNΣ was isolated using Puregene DNA purification kit (Qiagen) and the transposon insertion was verified by PCR with primers listed in Table S3. For all complementations, the respective gene and its native promoter were amplified from LAC chromosomal DNA using primers listed in Table S3. The product was digested with the respective restriction enzymes and ligated into the same restriction sites in pCM28. The resulting plasmid was electroporated into *E. coli* DC10B and sequencing was performed at the Molecular Biology Service Center at the University of Colorado Anschutz Medical Campus with chromosomal and vector primers (Table S3). The empty vector (pCM28) and the complementation vectors (pKAS23, pKAS25, pKAS33, pKAS34, pKAS35) were then electroporated into the respective mutant strain.

**References**

1. Crosby HA, Schlievert PM, Merriman JA, King JM, Salgado-Pabon W, Horswill AR. 2016. The Staphylococcus aureus Global Regulator MgrA Modulates Clumping and Virulence by Controlling Surface Protein Expression. PLoS Pathog 12:e1005604.

2. Schuster CF, Howard SA, Grundling A. 2019. Use of the counter selectable marker PheS* for genome engineering in Staphylococcus aureus. Microbiology 165:572-584.

3. Monk IR, Shah IM, Xu M, Tan MW, Foster TJ. 2012. Transforming the untransformable: application of direct transformation to manipulate genetically Staphylococcus aureus and Staphylococcus epidermidis. MBio 3.

4. Fey PD, Endres JL, Yajjala VK, Widhelm TJ, Boissy RJ, Bose JL, Bayles KW. 2013. A genetic resource for rapid and comprehensive phenotype screening of nonessential Staphylococcus aureus genes. MBio 4:e00537-12.

5. Olson ME. 2016. Bacteriophage Transduction in Staphylococcus aureus. Methods Mol Biol 1373:69-74.

6. Jacob AE, Hobbs SJ. 1974. Conjugal transfer of plasmid-borne multiple antibiotic resistance in Streptococcus faecalis var. zymogenes. J Bacteriol 117:360-72.

7. Boles BR, Thoendel M, Roth AJ, Horswill AR. 2010. Identification of genes involved in polysaccharide-independent Staphylococcus aureus biofilm formation. PLoS One 5:e10146.

8. Clewell DB, An FY, White BA, Gawron-Burke C. 1985. Streptococcus faecalis sex pheromone (cAM373) also produced by Staphylococcus aureus and identification of a conjugative transposon (Tn918). J Bacteriol 162:1212-20.

9. Bose JL, Fey PD, Bayles KW. 2013. Genetic tools to enhance the study of gene function and regulation in Staphylococcus aureus. Appl Environ Microbiol 79:2218-24.

10. Pang YY, Schwartz J, Thoendel M, Ackermann LW, Horswill AR, Nauseef WM. 2010. agr-Dependent interactions of Staphylococcus aureus USA300 with human polymorphonuclear neutrophils. J Innate Immun 2:546-59.
