# Supplementary material for: Processing, Export, and Identification of Novel Linear Peptides from Staphylococcus aureus
Source: mBio. 2020 Apr 14;11(2):e00112-20. doi: 10.1128/mBio.00112-20 (PMC7157817; doi:10.1128/mBio.00112-20)
Supplement: TABLE S2 [file mBio.00112-20-st002.docx]

**Table S2:** Bacterial strains/ plasmids

| **Strains/ Plasmids** | **Genotype** | **Reference** |
| --- | --- | --- |
| **Strains** | | |
| *E. faecalis* |  |  |
| JH2-2 | plasmid free, Rif^R^, Fus^R^ | 6 |
| *E. coli* |  |  |
| DC10B | Cloning strain (*dcm*-) | 3 |
| *S. aureus* |  |  |
| AH1263 | USA300 CA-MRSA, Erm^S^ (LAC*) | 7 |
| AH3559 | LAC* ∆*lgt* | This work |
| AH5582 | LAC* ∆*eep* | This work |
| AH5583 | LAC* ∆*camS* | This work |
| AH5584 | LAC* ∆*ecsAB* | This work |
| AH5585 | LAC* *lspA*::ΦNΣ, Erm^R^ | This work |
| **Plasmids** | | |
| pAM373::Tn*918* | responds to *staph*-cAM373, Tet^R^ | 8 |
| pJB38 | Mutation generation vector, Amp^R^ (*E.coli*), Cm^R^ (*S. aureus*) | 9 |
| pIMAY | Mutation generation vector, Cm^R^ (*E.coli, S. aureus*) | 3 |
| pCM28 | Expression vector, Amp^R^ (*E.coli*), Cm^R^ (*S. aureus*) | 10 |
| CFS246 | pJB38-*lgt* deletion vector, Amp^R^ (*E.coli*), Cm^R^ (*S. aureus*) | This work |
| pKAS07 | pJB38-*camS* deletion vector, Amp^R^ (*E.coli*), Cm^R^ (*S. aureus*) | This work |
| pKAS22 | pJB38-*ecsAB* deletion vector, Amp^R^ (*E.coli*), Cm^R^ (*S. aureus*) | This work |
| pKAS24 | pIMAY-*eep* deletion vector, Cm^R^ (*E.coli, S. aureus*) | This work |
| pKAS23 | pCM28-*camS*, Amp^R^ (*E.coli*), Cm^R^ (*S. aureus*) | This work |
| pKAS25 | pCM28-*ecsAB*, Amp^R^ (*E.coli*), Cm^R^ (*S. aureus*) | This work |
| pKAS33 | pCM28-*eep*, Amp^R^ (*E.coli*), Cm^R^ (*S. aureus*) | This work |
| pKAS34 | pCM28-*lgt*, Amp^R^ (*E.coli*), Cm^R^ (*S. aureus*) | This work |
| pKAS35 | pCM28-*lspA*, Amp^R^ (*E.coli*), Cm^R^ (*S. aureus*) | This work |

Rif^R^ - rifampicin resistant; Fus^R^ - fusidic acid resistant, Erm^S^ - erythromycin sensitive; Tet^R^ - tetracycline resistant, Amp^R^ - ampicillin resistant, Cm^R^ - chloramphenicol resistant
