# Supplementary figures and images for: Processing, Export, and Identification of Novel Linear Peptides from Staphylococcus aureus
Source: mBio. 2020 Apr 14;11(2):e00112-20. doi: 10.1128/mBio.00112-20 (PMC7157817; doi:10.1128/mBio.00112-20)

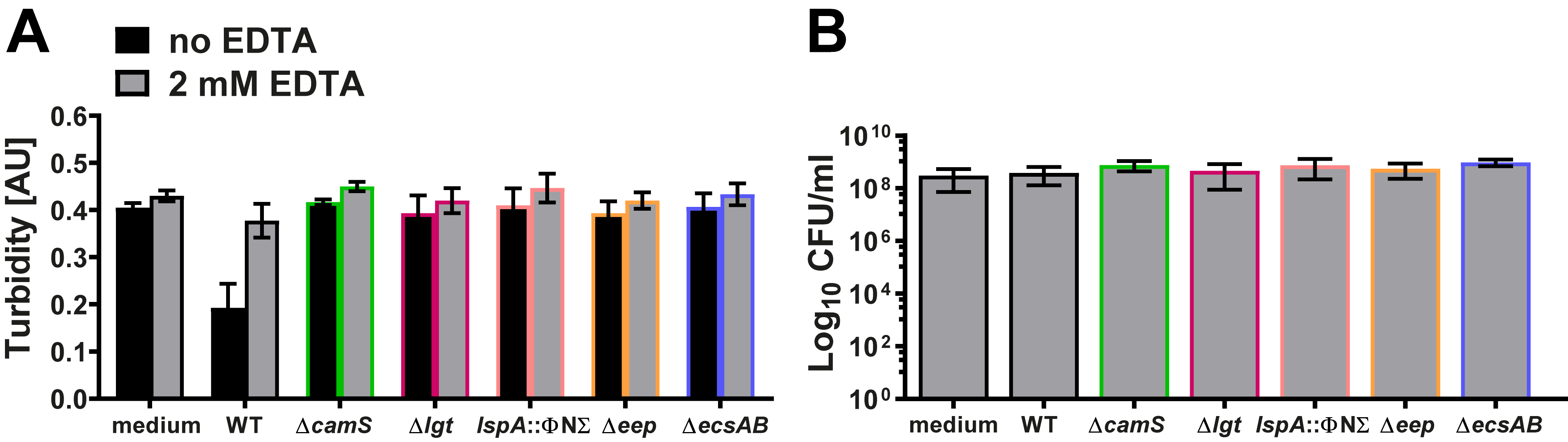

Supplement: FIG S1 [file mBio.00112-20-sf001.tif]

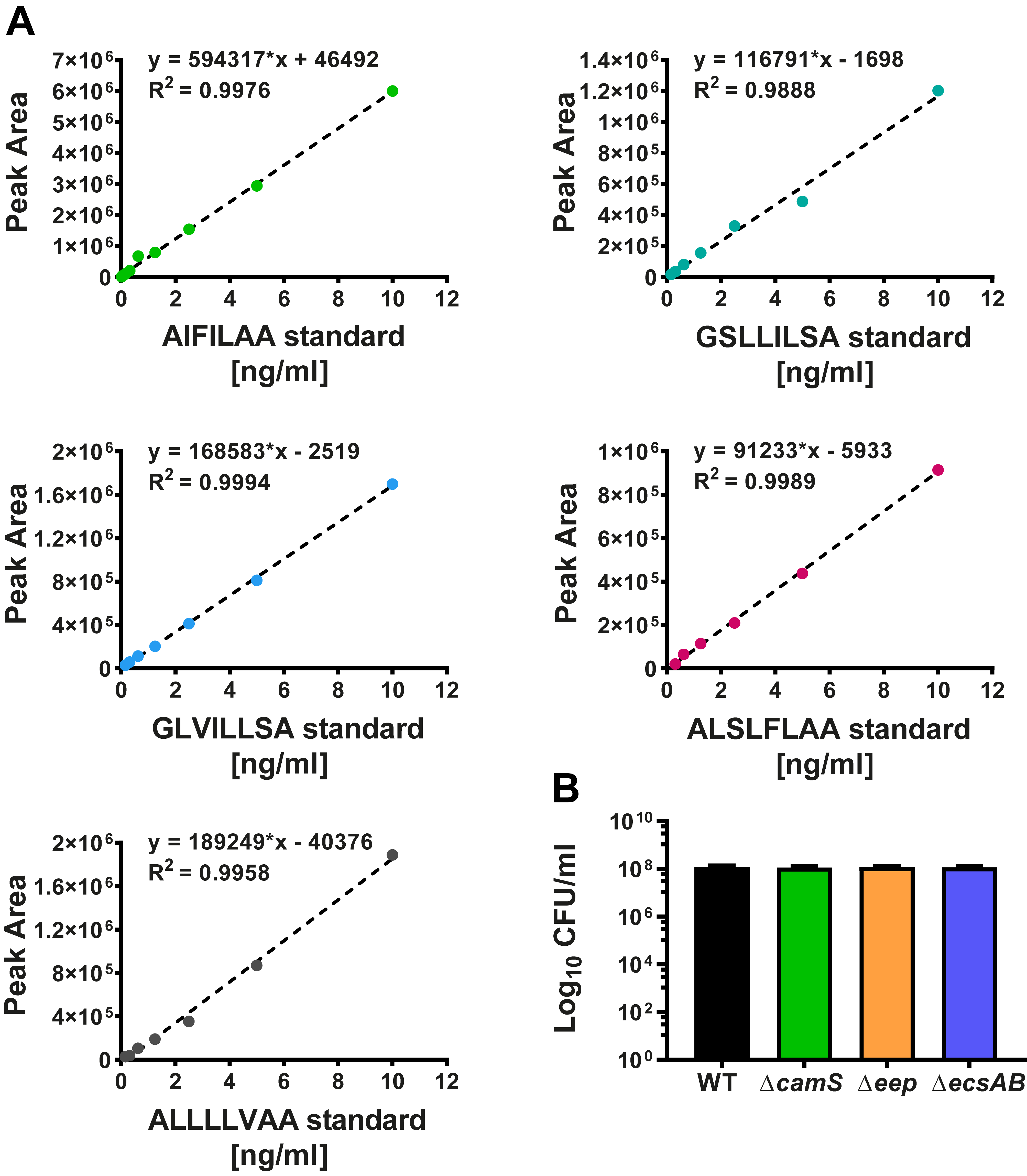

Supplement: FIG S2 [file mBio.00112-20-sf002.tif]
