# Supplementary material for: Processing, Export, and Identification of Novel Linear Peptides from Staphylococcus aureus
Source: mBio. 2020 Apr 14;11(2):e00112-20. doi: 10.1128/mBio.00112-20 (PMC7157817; doi:10.1128/mBio.00112-20)
Supplement: TABLE S3 [file mBio.00112-20-st003.docx]

**Table S3:** Primer list

| **Primer** | **5’ – 3’ sequence** | **Application** | **Reference** |
| --- | --- | --- | --- |
| KAS169 | TGCCACCTGACGTCTAAGAA | pJB38 PCR/ sequencing | This work |
| KAS170 | CCTCACATTTGTGCCACCTA | pJB38 PCR/ sequencing | This work |
| IM151 | TACATGTCAAGAATAAACTGCCAAAGC | pIMAY PCR/ sequencing | 3 |
| IM152 | AATACCTGTGACGGAAGATCACTTCG | pIMAY PCR/ sequencing | 3 |
| KAS113 | GAGTCAGTGAGCGAGGAAGC | pCM28 PCR/ sequencing | This work |
| KAS116 | GGAAACAAAAAGAGTATTTAGCTAGTG | pCM28 PCR/ sequencing | This work |
| CEF238 | GTTGTTGAATTCGACTACAAGTTTAATGAGTCGCTTAAC | *lgt* deletion | This work |
| CEF239 | **CACTACTTCAGTACTAGATCTTCAACCTA**CTCCTCACTCTTATGAC | *lgt* deletion | This work |
| CEF240 | **TAGGTTGAAGATCTAGTACTGAAGTAGTG**ATAGTTTGAGGAAATTTTTATCAA | *lgt* deletion | This work |
| CEF241 | GTTGTTGGTACCCTCGTTTATATTCTCTTTGTTTAAACTTCTGT | *lgt* deletion | This work |
| KAS47 | CCAGAAACGCCTGCAATTAT | *lgt* PCR/ sequencing | This work |
| KAS48 | GAATGCCTTGTTTGGTTCGT | *lgt* PCR/ sequencing | This work |
| KAS1 | TTCGAGCTCGGCCTGACTTTGACGAAGAT | *eep* deletion | This work |
| KAS2 | CCGGAATTCttaATAGCTCACTCGCTACACCTCG | *eep* deletion | This work |
| KAS3 | CCGGAATTCATTCCAACCAGCGAGTGAAC | *eep* deletion | This work |
| KAS4 | CGGGGTACCCGACTTCTGCTTTTTCGATGT | *eep* deletion | This work |
| KAS9 | ACGAGCTTCATCAACAAGGTTT | *eep* PCR/ sequencing | This work |
| KAS10 | GAACCAGGATTTGCACCAAC | *eep* PCR/ sequencing | This work |
| KAS11 | ACAGGGGCTTACTTGTTTGGTA | *eep* PCR/ sequencing | This work |
| KAS12 | AACGTCCTGATTCTTCCCATAA | *eep* PCR/ sequencing | This work |
| KAS26 | CGGAATTCTTAAGTATTGGACGAACAGGTG | *camS* deletion | This work |
| KAS27 | CCGCTCGAGttaCTTCATCGACATACCCCTCT | *camS* deletion | This work |
| KAS28 | CCGCTCGAGCCGCAAGTTCATATTTACAGT | *camS* deletion | This work |
| KAS29 | ACGCGTCGACGCGATGGTTATGGTCCCTAT | *camS* deletion | This work |
| KAS33 | TCAACAGGATGAGATGGGATT | *camS* PCR/ sequencing | This work |
| KAS34 | TCTGGTTTCGTATCTGGTGGT | *camS* PCR/ sequencing | This work |
| KAS41 | GGTGCATCGTTCAGTCCAC | *camS* PCR/ sequencing | This work |
| KAS42 | AACGGTAACTGAAGCGGAAT | *camS* PCR/ sequencing | This work |
| KAS68 | ACGCGTCGACTGTGATGTTTCTTCACCTTTG | *ecsAB* deletion | This work |
| KAS71 | GGGGTACCTGTGATCGAGAACGTAACCTG | *ecsAB* deletion | This work |
| KAS74 | CCGGGTACCTTGTACTCCTCGAGTGTACTGTGTTTGCAGTGACAT | *ecsAB* deletion | This work |
| KAS75 | CCGGTCGACTTGTACTCCTCGAGAAGGCACCTCCATGACTTATATT | *ecsAB* deletion | This work |
| KAS80 | CAACTCCGGCTGATGTGTAA | *ecsAB* PCR/ sequencing | This work |
| KAS81 | TCATCTCGTTTATTTGGCATTT | *ecsAB* PCR/ sequencing | This work |
| KAS86 | TCTGAACGCCGTATCATTTG | *ecsAB* PCR/ sequencing | This work |
| KAS87 | CCGAAAATTGTTTCTGACATGA | *ecsAB* PCR/ sequencing | This work |
| KAS37 | CCTCCTTCTCCTTTTTATTGGA | *lspA* Tn mutation check | This work |
| KAS38 | GCTGCATGGGGAATATTGAG | *lspA* Tn mutation check | This work |
| KAS84 | GTTGTTGGATCCTTAAGTATTGGACGAACAGGTG | *camS* complementation | This work |
| KAS85 | GTTGTTGTCGACAAGTTCGTGACATCGTTAGAGA | *camS* complementation | This work |
| KAS65 | CTGCTGCTGTTGAAGAAGGTAA | *camS* compl. sequencing | This work |
| KAS44 | CCCCTCTAGAAGAAAATGAAGATATTCGTGCTTTA | *camS* compl. sequencing | This work |
| KAS90 | GTTGTTGGATCCTTGCCGAAAATTGTTTCTGA | *ecsAB* complementation | This work |
| KAS91 | GTTGTTGTCGACTCTGAACGCCGTATCATTTG | *ecsAB* complementation | This work |
| KAS202 | TAAACACCCAATAATTGCTGTCA | *ecsAB* compl. sequencing | This work |
| KAS203 | TCATTTTTCTAAGGGGATGAAA | *ecsAB* compl. sequencing | This work |
| KAS156 | GTTGTTGGATCCTAGTGGAATCAGGATTTAAGC | *eep* complementation | This work |
| KAS157 | GTTGTTCTGCAGttaccGCTTCATAATTATTTATCCTCCT | *eep* complementation | This work |
| KAS171 | GGAATCCGAGAACATACTTCGT | *eep* compl. sequencing | This work |
| KAS172 | AAACAATCGAGGTGTAGCGAGT | *eep* compl. sequencing | This work |
| KAS159 | GTTGTTGTCGACttagCCTGTTTGTTCTTTATCTGTAATGT | *lspA* complementation | This work |
| KAS160 | GTTGTTGGATCCCAGCCTGCTTTCCTAATTT | *lspA* complementation | This work |
| KAS93 | GTTGTTGTCGACCCGCCAACTAATTCCAATATATCA | *lgt* complementation | This work |
| KAS94 | GTTGTTGGATCCGGGTTCACCTCAATTGTATTTATCC | *lgt* complementation | This work |
| KAS95 | GTTGTTGGATCCATGGGTATTGTATTTAACTATATAGATC | *lgt* complementation | This work |
| KAS96 | GTTGTTGAATTCCTCAAACTATCACTACTTCACTTT | *lgt* complementation | This work |
| KAS173 | TGGTGGTTTAATAGGTGGCTTT | *lgt* compl. sequencing | This work |

overlapping nucleotides are in bold; restriction enzyme sites are underlined
